# Understanding Patient Decision-Making in Breast Cancer Surgery: Risk Perception, Communication, and Psychosocial Influences

**DOI:** 10.3390/medsci13040225

**Published:** 2025-10-09

**Authors:** Eman Sbaity, Tasnim Diab, Jana Haroun, Nagham Ramadan, Ghina Khalil, Nathalie Chamseddine, Rawan Diab, Hadi Mansour, Mohyeddine El Sayed, Maya Charafeddine, Jaber Abbas, Hazem I. Assi

**Affiliations:** 1 Department of General Surgery, American University of Beirut Medical Center, Beirut P.O. Box 11-0236, Lebanon; es25@aub.edu.lb (E.S.); ja19@aub.edu.lb (J.A.); 2 Department of Internal Medicine, Division of Hematology and Oncology, Naef K Bassile Cancer Institute, American University of Beirut Medical Center, Beirut P.O. Box 11-0236, Lebanon; td19@aub.edu.lb (T.D.); nagham_ramadan@hotmail.com (N.R.); nc39@aub.edu.lb (N.C.); mc16@aub.edu.lb (M.C.); 3 Faculty of Medicine, American University of Beirut, Beirut P.O. Box 11-0236, Lebanon; joh02@mail.aub.edu (J.H.); gik03@mail.aub.edu (G.K.); rd74@aub.edu.lb (R.D.); hym09@mail.aub.edu (H.M.); mke29@mail.aub.edu (M.E.S.)

**Keywords:** mastectomy, contralateral prophylactic mastectomy, decision-making, risk communication, surgical methods

## Abstract

**Background:** Despite evidence discouraging contralateral prophylactic mastectomy (CPM) in average-risk patients, its use is increasing globally. While well-studied in Western settings, little is known about the factors influencing CPM decisions in the Middle East and North Africa (MENA) region. This study explores clinical, psychosocial, and communication-related factors associated with CPM choices among women with early-stage breast cancer. **Methods:** We conducted a retrospective study of 253 early-stage breast cancer patients who underwent mastectomy, with or without CPM, at the American University of Beirut Medical Center. Clinical and demographic data were extracted from medical records, and decision-making factors were assessed through tailored patient questionnaires. Associations were analyzed using chi-square tests and multivariable logistic regression. **Results:** Of the 253 women included in the study, 37 underwent CPM, while 216 had unilateral mastectomy (UM). Compared to the UM group, women who chose CPM were more likely to have a college education (96.9% vs. 57.6%, *p* < 0.001), be employed (69.7% vs. 41.3%, *p* = 0.002), and report a family history of breast cancer (55.6% vs. 30.2%, *p* = 0.003). Immediate reconstruction was significantly more common among CPM patients (67.6% vs. 16.4%, *p* < 0.001), and the 30-day rehospitalization rate was also higher (16.2% vs. 6.1%, *p* = 0.031). Women in the CPM group were more likely to prioritize extending life (84.6% vs. 56.7%, *p* = 0.007) and achieving peace of mind (80.8% vs. 49.3%, *p* = 0.003). Although all CPM patients cited risk reduction as a primary motivator, only 46.2% believed they had a lower recurrence risk than their peers (vs. 20% of UM patients, *p* < 0.001). Decisions to undergo UM were more frequently influenced by physicians’ recommendations (95.3% vs. 53.8%, *p* < 0.001), whereas CPM decisions appeared to be more patient-driven. Additionally, CPM patients reported more negative expectations and higher dissatisfaction with pain (57.7% vs. 32.0%, *p* = 0.012) and reconstructive outcomes (54.5% vs. 27.5%, *p* = 0.035). **Conclusions:** In this first study from the MENA region exploring CPM decision-making, choices were largely driven by personal preferences rather than clinical risk. These findings highlight the need for improved risk communication, shared decision-making, and broader integration of genetic counseling in surgical planning.

## 1. Introduction

Breast cancer is the most prevalent cancer among women worldwide, with a substantial number of cases being diagnosed at an early stage [1]. Over the past few decades, the implementation of routine mammography screening allowed the early detection of breast tumors, even in instances where they are small and not palpable [2,3]. In Lebanon, breast cancer stands as the predominant type of cancer, accounting for approximately 37% of cancer diagnoses in females and 20% of all cancer cases in the country [4]. In this context, breast cancer represents a major public health burden both globally and regionally and understanding treatment choices has important implications for survival and quality of life.

The management of early-stage breast cancer involves a complex array of treatment choices, including breast-conserving surgery and different mastectomy techniques. Contralateral prophylactic mastectomy (CPM) refers to the surgical removal of the unaffected breast in patients diagnosed with unilateral breast cancer, typically with the intention of reducing the likelihood of contralateral breast cancer (CBC) [5]. However, not all women pursue CPM solely to reduce CBC risk; other motivations include eliminating the need for future screening, achieving breast symmetry, and maximizing reconstructive options, particularly when access to complex procedures like DIEP flap reconstruction is limited to a single surgical opportunity [6].

Despite the limited clinical indications for CPM, its uptake has been steadily increasing worldwide. In recent years, there has been an increasing inclination towards more aggressive surgical approaches, including ipsilateral mastectomy, often accompanied by the option of CPM. This trend is observed even in cases where the disease is unilateral and localized [7].

Among women diagnosed with unilateral breast cancer, the average annual probability of developing CBC is approximately 0.4%. This leads to a cumulative incidence of 1.9% over a five-year period [7,8].

The current guidelines provided by the National Comprehensive Cancer Network and the American Society of Breast Surgeons reserve considering CPM for individuals at an increased risk of CBC, such as those harboring BRCA1/2 mutations or individuals with a notable family history [9,10,11]. However, Hawley et al. reported that merely 31% of women opting for CPM have a BRCA mutation or a significant family history [12]. These findings suggest that the decision to undergo CPM may not always align with an elevated risk of developing CBC. This implies variations in adherence to established guidelines, emphasizing the importance of having a comprehensive understanding of the various factors influencing patients’ decisions when considering CPM. This paradox of increasing CPM rates despite limited indications highlights the need to explore not only clinical drivers but also psychosocial, cultural, and communication factors that influence surgical choices.

Many large randomized clinical trials have demonstrated that the long-term survival rates subsequent to lumpectomy are equivalent to those observed following mastectomy [13,14]. Furthermore, a multitude of studies utilizing various methodological approaches have unequivocally demonstrated that there is minimal to no overall survival advantage linked to CPM [15,16]. Psychosocial motivations such as fear of recurrence, desire for peace of mind, and body image concerns have been consistently identified as important contributors to CPM decisions in Western literature [17,18]. However, it is unclear whether these same drivers play a similar role in Middle Eastern populations, where cultural norms, family involvement, and healthcare system differences may shape decisions in unique ways.

The decision-making process regarding the extent of surgical intervention in early-stage breast cancer is intricate and influenced by multiple factors including oncologic, psychological, and sociodemographic factors [19]. Understanding the determinants influencing the selection of ipsilateral mastectomy, with or without CPM, is of paramount importance. This not only facilitates personalized patient care but also plays a pivotal role in shaping healthcare policies and optimizing the overall prognosis and satisfaction of breast cancer survivors.

Although mastectomy is generally a safe surgical procedure with infrequent instances of major complications, it gives rise to aesthetic considerations that can impact the long-term overall quality of life. The growing inclination towards reconstructive surgery introduces a level of intricacy, which calls for meticulous consideration of potential prolonged recovery periods and the likelihood of additional complications [20].

While the decision-making process surrounding CPM has been well-studied in the United States and other Western countries, there remains a notable lack of region-specific data from the Middle East and North Africa (MENA) region. This study aims to explore a range of determinants, including patient, physician, and tumor-related factors, to gain a deeper insight into the decision-making process. Furthermore, we intend to assess patients’ satisfaction levels and investigate the incidence of postoperative complications, with the aim of achieving a comprehensive understanding of the overall experience for patients undergoing different surgical choices. To our knowledge, this is the first study from the MENA region to investigate CPM decision-making, thereby addressing an important regional research gap.

## 2. Materials and Methods

### 2.1. Study Design

This is a retrospective study involving all patients with early-stage or locally advanced unilateral breast cancer who underwent ipsilateral mastectomy, with or without CPM, at the American University of Beirut Medical Center (AUBMC) between 1 January 2012 and 31 December 2018. The study was conducted according to the ethical principles stated in the Declaration of Helsinki (2013). The Institutional Review Board (IRB) at AUBMC reviewed the study proposal, and approval from the IRB was granted prior to data collection.

### 2.2. Participants

The inclusion criteria consisted of patients with early-stage or locally advanced unilateral breast cancer, non-metastatic, with or without regional lymph node involvement, and falling within the T0–T3 classification (Stage I, Stage IIA, Stage IIB, and Stage IIIA breast cancers). T4 tumors were ineligible for consideration, along with patients diagnosed with metastatic disease or those with a history of recurrence.

After analyzing the records of 562 patients and excluding those with metastatic disease, individuals who underwent breast-conserving surgery and those experiencing a recurrence of the disease, our study focused on a sample of 253 patients from whom we collected data. Of these, 216 underwent (UM), while 37 opted for CPM, resulting in a 14.6% rate of CPM among women diagnosed with early-stage or locally advanced breast cancer.

We successfully contacted 176 participants (70%) for the administration of the questionnaire. Among the respondents, 150 had undergone UM and 26 had undergone CPM. On average, the survey was completed five years after surgery (range 2–8 years).

### 2.3. Measurements

We divided the study into two phases. The initial phase involved a comprehensive review of charts, including baseline socio-demographic information, disease characteristics, and details about disease treatment. We conducted data retrieval through the Electronic Medical Record system at AUBMC, using patients’ identification numbers.

The second phase consisted of a questionnaire comprising 23 items that focused on decision-making, knowledge, risk perceptions, and concerns related to breast cancer. We administered this questionnaire via phone calls and featured two versions: one tailored for individuals who had undergone UM and the other for those who had chosen CPM. The framework of the questionnaire was adapted from a study conducted by Rosenberg et al., titled “Perceptions, knowledge, and satisfaction with CPM among young women with breast cancer: A cross-sectional survey” [21]. The full questionnaires for both UM and CPM groups are provided in Supplementary Materials S1 and S2.

Participants were subsequently asked about their motivations for choosing mastectomy, which included cosmetic, preventive, genetic, or other reasons. Additionally, the questionnaire included inquiries about patient satisfaction with the surgery, covering aspects such as cosmetic outcomes, pain, numbness, and any reconstruction performed.

### 2.4. Statistical Analysis

We described continuous variables as means ± standard deviations or median (Interquartile range), while categorical variables were described as frequencies and percentages. For inferential statistics, we evaluated variables showing significant associations with the decision to undergo UM or CPM using the Chi-square test for categorical variables and the t-test for continuous variables. In addition, multivariable logistic regression analysis was performed to identify independent predictors of choosing CPM, with results reported as odds ratios (ORs) and 95% confidence intervals (CIs). All *p*-values were two-sided, and a significance level of *p* < 0.05 was applied to all analyses. The statistical analyses were performed using the SPSS version 29.0 statistical package.

## 3. Results

### 3.1. Study Population Characteristics

Out of 253 patients included in the study, 216 (85.4%) underwent (UM) and 37 (14.6%) underwent contralateral prophylactic mastectomy (CPM). Among the 176 patients who completed the questionnaire, 150 had UM and 26 had CPM.

The median age at diagnosis for the overall cohort was 50 years (26–85). Patients who underwent UM had a mean age of 52 ± 12 years, while those who opted for CPM had a significantly lower mean age of 44 ± 10 years at diagnosis (*p* < 0.001). Women who chose CPM as compared to those who did not, had significantly higher levels of education (57.6% vs. 96.9%, *p* < 0.001) and were more likely to be employed (41.3% vs. 69.7%, *p* = 0.002), urban residents (65.6% vs. 86.1%, *p* = 0.014), and have a family history of breast cancer (30.2% vs. 55.6%, *p* = 0.003) (Table 1).

In total, BRCA testing data were available for 56 patients, constituting 22.1% of the study population. Noteworthy differences in testing rates were observed between the CPM and UM groups. A total of 54.1% of CPM recipients underwent testing, compared to only 16.7% of those undergoing UM (*p* < 0.001). Among individuals with identifiable BRCA testing results, only six patients had positive findings. The prevalence of confirmed BRCA-positive status did not show a statistically significant difference between patients who underwent CPM and those who opted for UM (15% vs. 8.3%, *p* = 0.655). However, no conclusions can be made because of the large scale of missing data.

Significant differences were observed in tumor size, with patients having smaller tumors opting for CPM. Additionally, variations were noted in preferences for immediate reconstruction and choices related to breast reconstruction between the two groups. Furthermore, a significant difference (*p* = 0.031) in the 30-day rehospitalization rate was observed, with a higher incidence in the CPM group. No significant differences were noted between both cohorts in terms of marital status, quadrant involvement, multifocality, multicentricity, histopathologic type, tumor grade, or HER2 status (Table 1).

### 3.2. Decision Making

A greater proportion of women who chose CPM regarded extending life as a crucial factor compared to those opting for UM (84.6% vs. 56.7%, *p* = 0.007). Similarly, recipients of CPM deemed peace of mind to be most important (80.8% vs. 49.3%, *p* = 0.003) (Table 2).

While advice from friends or family and having a strong family history of breast cancer did not significantly affect patient decisions regarding whether to undergo CPM or not, a known genetic mutation influenced patients in favor of choosing CPM (100% vs. 62.5%). Notably, following the doctor’s recommendation was significantly more crucial for individuals who opted for UM (95.3% vs. 53.8%, *p* < 0.001). This finding is reflected in responses to the question, “who first brought up the idea of having your breast(s) removed.” Notably, 78.7% of patients undergoing UM attributed the idea to their oncologist or surgeon. In contrast, 80% of those who chose to undergo CPM stated that it was their own decision (*p* < 0.001).

A significant aspect of the decision-making process for patients opting for CPM is that 65.4% of them prioritized the desire to have both breasts look the same post-surgery. Only 34.6% reported that the desire to enhance the aesthetics of their breasts was a significant factor in their decision. Remarkably, all patients opting for CPM cited a crucial motivation to reduce the risk of developing CBC. Moreover, 65.4% expressed no worry regarding the potential limitations of screening in detecting cancer in the opposite breast as a rationale for choosing CPM.

Furthermore, all patients undergoing CPM acknowledged feeling an increased risk of developing CBC in the future. Additionally, only 19.2% had an abnormal imaging of the contralateral breast prior to surgery.

While the above findings highlight key clinical and psychosocial influences, a multivariable approach was used to identify independent predictors of CPM. Table 3 presents the results of this logistic regression analysis. Women with a known BRCA mutation had significantly higher odds of undergoing CPM compared to those without a mutation (OR = 4.96, 95% CI: 1.43–17.21, *p* = 0.012). Immediate breast reconstruction also emerged as a strong independent predictor (OR = 7.73, 95% CI: 2.43–24.59, *p* = 0.001). Among psychosocial factors, the desire to follow the physician’s recommendation was associated with significantly lower odds of choosing CPM (OR = 0.036, 95% CI: 0.008–0.172, *p* < 0.001), while peace of mind was positively associated with the decision (OR = 4.76, 95% CI: 1.01–22.38, *p* = 0.048). In contrast, family history, advice from others, and perceived survival benefit were not statistically significant.

### 3.3. Decision Quality and Communication

Beyond clinical and psychosocial motivators, we assessed patient-reported decision quality and the extent of physician–patient communication at the time of surgical decision-making (Table 4). Overall, satisfaction and confidence in the chosen surgical path were high in both groups, with mean confidence scores of 9.05 ± 1.5 for CPM and 9.35 ± 1.2 for UM (*p* = 0.349). Similarly, the vast majority of patients in both groups indicated that they would make the same decision again (92.3% CPM vs. 94% UM; *p* = 0.669).

When exploring decision quality, comparable proportions in each group felt well-informed about the risks and benefits (80.8% CPM vs. 78% UM; *p* = 0.751), but a significantly higher proportion of patients in the CPM group reported being clear about which benefits and risks mattered most to them (100% vs. 80%, *p* = 0.009). Most patients felt they had adequate support and advice (73.1% CPM vs. 82.7% UM; *p* = 0.277) and were sure about the best choice for them (88.5% CPM vs. 88.7% UM; *p* = 1.00).

Reported communication with physicians was comparable between groups. Discussions about the reasons to undergo surgery were noted by 38.5% of CPM patients and 43.4% of UM patients (*p* = 0.643), while discussions about reasons not to proceed were reported by 69.2% and 62.7%, respectively (*p* = 0.521). Although not statistically significant, a higher proportion of CPM patients reported discussing concerns about the risk of contralateral breast cancer (88.5% vs. 73.8%; *p* = 0.107).

When comparing expectations versus experiences with surgery (Table 5), there was no statistically significant difference in reported cosmetic outcomes between the groups (*p* = 0.118). A higher percentage of CPM patients reported worse-than-expected pain (57.7% vs. 32.0%, *p* = 0.012), more unexpected surgeries (34.6% vs. 14.7%, *p* = 0.023), and more complications from reconstruction (54.5% vs. 27.5%, *p* = 0.035). CPM patients were also more likely to report negative experiences with tissue expander filling (68.8% vs. 32.4%, *p* = 0.016).

Notably, there was no statistically significant difference observed in the occurrence of wound complications between both groups, with 10.8% in the CPM group compared to 13% in the UM group (*p* = 0.796).

### 3.4. Breast Cancer Risk Knowledge and Perception

A significant difference was observed in the perception of breast cancer mortality between both groups. In the UM group, 88.6% of participants believed that most women with early-stage breast cancer would die from other causes, while in the CPM group, only 53.8% held the same belief (*p* < 0.001) (Table 6).

Regarding the risk of developing CBC without CPM, UM patients estimated that, on average, 18 ± 15 out of 100 women would develop cancer in the contralateral breast within 5 years. In contrast, those who underwent CPM estimated that 35 ± 20 out of 100 women would develop cancer in the other breast within the same period (*p* = 0.001).

### 3.5. Breast Cancer Worry

Worry about developing CBC in the future was significantly more prevalent among CPM patients (84.6%) compared to those in the UM group (61.3%, *p* = 0.022).

Participants were also asked to compare their perceived risk of cancer recurrence to that of other women with early-stage breast cancer. A significantly higher proportion of CPM patients believed their risk was greater than that of their peers (30.8% vs. 14.7%, *p* < 0.001).

## 4. Discussion

This study provides the first in-depth analysis of CPM decision-making among women with early-stage breast cancer in the MENA region. Our findings reveal a pattern of deviation from evidence-based guidelines, as many women opted for CPM based on subjective motivations rather than high-risk clinical indications. These included the desire for peace of mind, the belief that CPM would extend survival, and cosmetic preferences. Decisions appeared to be more predominantly patient-driven, with physician recommendations playing a limited role, suggesting a shift in decision-making autonomy, with potential clinical implications.

International studies have consistently reported rising rates of CPM, particularly among younger and more educated women [22,23,24,25]. Similar drivers such as emotional reassurance, perceived risk reduction, and body image concerns have been cited globally [17,18]. Our results align with these trends but uniquely contextualize them within a Middle Eastern population, where cultural norms and healthcare dynamics differ. In our cohort, women cited peace of mind, survival expectations, and symmetry as key motivators for CPM, even in the absence of objective clinical risk.

Previous research shows that few women undergoing CPM complete BRCA testing, and among those who do, confirmed pathogenic mutations are uncommon [24,26]. Despite access to genetic counseling at a tertiary academic center, testing in our cohort remained underutilized: only slightly more than half of CPM patients were tested, and few had pathogenic variants. In Lebanon, BRCA testing is costly and often not covered by insurance, which likely explains much of the missing data. This gap limits opportunities for risk-based counseling and shows the challenges of using genetic testing in surgical planning in resource-limited settings, highlighting the need to include such testing more regularly into preoperative decision-making whenever we can.

In the Lebanese and broader MENA cultural context, family members often have a strong influence on medical decisions, even when patients feel the choice is their own. In addition, the limited availability and high cost of genetic testing, along with variability in access to screening and reconstructive services, reflect system-level challenges that likely shaped decision-making in our cohort. Cultural values that emphasize femininity, motherhood, and body image may increase the importance women place on reconstruction and breast symmetry. At the same time, limited public health awareness and unequal access to screening can fuel anxiety and lead to an overestimation of contralateral breast cancer risk.

These findings point to an urgent need to enhance shared decision-making practices in Lebanon. The predominance of personal preference over clinical guidance may reflect a communication gap, where patients may not fully grasp the risks, benefits, or lack of survival advantage associated with CPM [27,28]. This points to a broader issue of inadequate risk communication, where clinical recommendations may be underemphasized, misunderstood, or dismissed. Ensuring that patients are adequately informed, particularly about the lack of survival benefit from CPM in most cases, is critical to safeguarding patient autonomy while preventing overtreatment [15,16]. Because the survey was completed, on average, five years after surgery, the reported responses likely reflect long-term perceptions of satisfaction, although the study was not specifically designed as a longitudinal follow-up.

Our findings also showed clear misperceptions about both survival benefit and recurrence risk. These misperceptions are consistent with international reports and highlight an important target for intervention, as they may contribute more to surgical decisions than objective clinical risk. Many women believed that CPM would improve survival, even though evidence does not support this. Others overestimated their personal risk of contralateral breast cancer or recurrence compared to peers. These gaps in understanding point to the need for better counseling and shared decision-making tools to help patients have a more realistic view of risks and benefits when considering CPM.

In the Lebanese setting, where access to reconstructive surgery and patient education can vary widely, a refined and culturally sensitive counseling approach is vital. The higher rates of CPM among urban, educated, and employed women may reflect greater access to care but also a higher likelihood of exposure to misconceptions and emotional drivers [25,29]. This pattern reveals the complex interplay between empowerment and misinformation and the importance of targeted educational tools that balance emotional needs with evidence-based care.

Based on these insights, we propose several actionable recommendations. First, structured multidisciplinary counseling including oncology, surgery, and genetics is needed to support high-quality decisions [19]. Second, culturally tailored decision aids could be developed by integrating region-specific values, using plain language and visuals, incorporating Arabic-language narratives, and reflecting local healthcare realities. In clinical settings, such tools could be delivered through multidisciplinary counseling sessions and made available in oncology clinics and community health programs to support informed, patient-centered decision-making [17]. Third, public and professional education campaigns are necessary to address persistent misconceptions about CPM. Finally, more research should explore the long-term psychosocial and quality-of-life impacts of CPM in Middle Eastern settings [30,31,32].

Our study has several limitations. The relatively small sample size may limit generalizability, and as with all retrospective surveys, there is a risk of selection and recall bias, particularly given the five-year interval from surgery to survey completion. As a tertiary referral center, some patients returned abroad after surgery and could not be reached, which may have contributed to selection bias. In addition, the questionnaire, though adapted from prior studies, was not validated and did not capture certain factors such as financial considerations that may influence decision-making. The regression analysis should also be interpreted with caution, as the small CPM subgroup resulted in wide confidence intervals and exploratory rather than definitive associations. Despite these limitations, this remains the first study from the MENA region to capture surgical decision-making among breast cancer patients.

## 5. Conclusions

While CPM remains clinically appropriate for a small subset of high-risk patients, our findings suggest that among women with low clinical risk in Lebanon, the decision is often associated with emotional reassurance and perceived benefits rather than evidence-based recommendations. This reflects a growing trend toward patient-driven decision-making. Aligning patient expectations with medical evidence is essential to promote appropriate surgical choices.

## Figures and Tables

**Table 1:** Patient and tumor characteristics.

	UM N (%)	CPM N (%)	*p*-Value
**Marital Status**			
Single	47 (21.8)	5 (13.5)	0.251
Married	169 (78.2)	32 (86.5)	
**College Education**			
Yes	114 (57.6)	31 (96.9)	<0.001
No	84 (42.4)	1 (3.1)	
**Employment**			
Yes	86 (41.3)	23 (69.7)	0.002
No	122 (58.7)	10 (30.3)	
**Medical Insurance**			
Private	117 (83.5)	33 (89.2)	0.379
Public	35 (16.5)	4 (10.8)	
**Residence**			
Urban	137 (65.6)	31 (86.1)	0.014
Rural	72 (34.4)	5 (13.9)	
**Family History of Breast Cancer**			
Yes	62 (30.2)	20 (55.6)	0.003
No	143 (69.8)	16 (44.4)	
**Tumor size**			
**DCIS**	17 (7.9)	6 (16.2)	0.014
T1	89 (41.4)	21 (56.8)	
T2–T3	109 (50.7)	10 (27)	
**Quadrant**			
Central	61 (28.6)	13 (35.1)	0.424
Non-central	152 (71.4)	24 (64.9)	
**Multifocal**			
Yes	65 (30.2)	12 (32.4)	0.788
No	150 (69.8)	25 (67.6)	
**Multicentric**			
Yes	38 (17.7)	10 (27)	0.181
No	177 (82.3)	27 (73)	
**Histopathologic Type**			
DCIS	17 (7.9)	6 (16.2)	0.261
IDC	161 (74.5)	26 (70.3)	
ILC	19 (8.8)	4 (10.8)	
Others	19 (8.8)	1 (2.7)	
**Grade**			
Grade 1–2	126 (59.7)	23 (62.2)	0.875
Grade 3	85 (40.3)	14 (37.8)	
**Molecular Subtype**			
Luminal A	28 (18)	3 (11.5)	0.227
Luminal B HER2 −	42 (27)	12 (46.1)	
Luminal B HER2 +	37 (23.8)	6 (23)	
HER2-neu non-luminal	22 (14.2)	4 (15)	
Basal-like	26 (16.7)	1 (3.8)	
**Nodal Status**			
N0	133 (62.4)	21 (56.8)	0.512
N ≥ 1	80 (37.6)	16 (43.2)	
**BRCA Mutation**			
No mutation	33 (15.3)	17 (45.9)	<0.001
BRCA1/BRCA2 +	3 (1.4)	3 (8.1)	
Not Tested	180 (83.3)	17 (45.9)	
**Immediate Reconstruction**			
Yes	35 (16.4)	25 (67.6)	<0.001
No	179 (83.6)	12 (32.4)	
**Breast Reconstruction**			
Yes	55 (26.4)	30 (81.1)	<0.001
No	153 (73.6)	7 (18.9)	
**30 days Rehospitalization**			
Yes	13 (6.1)	6 (16.2)	0.031
No	201 (93.9)	31 (83.3)	
**Therapy Received**			
**Chemotherapy**			
None	69 (32.5)	15 (41.7)	0.555
Adjuvant	105 (49.5)	16 (44.4)	
Neoadjuvant	38 (17.9)	5 (13.9)	
**Adjuvant Radiotherapy**			
Yes	75 (35)	14 (37.8)	0.743
No	139 (65)	23 (62.2)	

CPM, contralateral prophylactic mastectomy; UM, unilateral mastectomy.

**Table 2:** Significance of factors considered by women when choosing UM or CPM.

	UM N (%)	CPM N (%)	*p*-Value
**Desire to lower the chance of recurrence in “the same breast”**			
**Important**	109 (72.7%)	22 (84.6%)	0.197
**Not important**	41 (27.3%)	4 (15.4%)	
**Desire to prevent breast cancer from spreading to other places in the body**			
**Important**	114 (76%)	24 (92.3%)	0.062
**Not important**	36 (24%)	2 (7.7%)	
**Desire to improve survival/ extend life**			
**Important**	85 (56.7%)	22 (84.6%)	0.007
**Not important**	65 (43.3%)	4 (15.4%)	
Desire for peace of mind *			
**Important**	74 (49.3%)	21 (80.8%)	0.003
**Not important**	76 (50.7%)	5 (19.2%)	
**Advice from friends or family**			
**Important**	74 (49.3%)	11 (42.3%)	0.508
**Not important**	76 (50.7%)	15 (57.7%)	
**Have strong family history of breast cancer**			
**Important**	29 (67.4%)	9 (81.8%)	0.474
**Not important**	14 (32.6%)	2 (18.2%)	
**Have known genetic change such as a BRCA1 or BRCA 2**			
**Important**	5 (62.5%)	2 (100%)	0.467
**Not important**	3 (37.5%)	0	
Desire to follow Dr’s recommendation **			
**Important**	143 (95.3%)	14 (53.8%)	<0.001
**Not important**	7 (4.7%)	12 (46.2%)	

CPM, contralateral prophylactic mastectomy; UM, unilateral mastectomy; * single most important reason to undergo CPM (27%); ** single most important reason to undergo UM (32%).

**Table 3:** Multivariable logistic regression analysis of clinical and psychosocial predictors of CPM.

Variable	Odds Ratio (OR)	95% CI for OR	*p*-Value
**BRCA mutation status**			
BRCA+ vs. negative	4.961	1.430–17.209	0.012
Untested vs. negative	1.073	0.053–21.614	0.963
Family history of breast cancer	0.606	0.179–2.056	0.422
Immediate reconstruction	7.729	2.430–24.585	0.001
Desire to follow doctor’s recommendation	0.036	0.008–0.172	<0.001
Desire for peace of mind	4.757	1.011–22.380	0.048
Desire to improve survival/extend life	1.370	0.301–6.233	0.684
Advice from friends/family	0.576	0.174–1.899	0.364

The logistic regression model was statistically significant (χ^2^ = 63.4, df = 8, *p* < 0.001), explaining 30.4% (Cox and Snell R^2^) to 53.5% (Nagelkerke R^2^) of the variance in CPM choice, with an overall classification accuracy of 90.3%.

**Table 4:** Decision-making and physician communication in UM vs. CPM patients.

	UM N (%)	CPM N (%)	*p*-Value
**When making the decision, did you feel you knew the benefits and risks of each option?**			
Yes	117 (78%)	21 (80.8%)	0.751
No	33 (22%)	5 (19.2%)	
**Were you clear about which benefits and risks mattered most to you?**			
Yes	120 (80%)	26 (100%)	0.009
No	30 (20%)	0 (0%)	
**Did you have enough support and advice to make a choice?**			
Yes	124 (82.7%)	19 (73.1%)	0.277
No	26 (17.3%)	7 (26.9%)	
**Did you feel sure about the best choice for you?**			
Yes	133 (88.7%)	23 (88.5%)	1.00
No	17 (11.3%)	3 (11.5%)	
**How much did you and your doctor talk about the reasons for having your breast(s) removed?**			
A lot	65 (43.4%)	10 (38.5%)	0.643
A little/Not at all	85 (56.7%)	16 (61.5%)	
**How much did you and your doctor talk about the reasons not to have your breast(s) removed?**			
A lot	94 (62.7%)	18 (69.2%)	0.521
A little/Not at all	56 (37.3%)	8 (30.8%)	
**How much did you and your doctor talk about how you felt about the possibility that cancer might occur in the other breast?**			
A lot	110 (73.8%)	23 (88.5%)	0.107
A little/Not at all	39 (26.2%)	3 (11.5%)	
**On a scale from 0 to 10, how confident are you that removing your breast(s) was the right decision?**	9.35 ± 1.2	9.05 ± 1.5	0.349
**If you could make this decision again, would you still choose to have your breast(s) removed?**			
Yes	140 (94%)	24 (92.3%)	0.669
No	9 (6%)	2 (7.7%)	

**Table 5:** Women’s reported experiences in relation to expectations associated with UM or CPM.

	UM N (%)	CPM N (%)	*p*-Value
**Cosmetic results**			
Worse than expected	67 (45)	16 (61.5)	0.118
Better than expected	82 (55)	10 (38.5)	
**Pain at surgical site**			
Worse than expected	48 (32)	15 (57.7)	0.012
Better than expected	102 (68)	11 (42.4)	
**Numbness/tingling in chest**			
Worse than expected	47 (31.8)	9 (34.6)	0.774
Better than expected	101 (68.2)	17 (65.4)	
**Number of surgeries/procedures**			
Worse than expected	22 (14.7)	9 (34.6)	0.023
Better than expected	128 (85.3)	17 (65.4)	
**Amount of follow up imaging or tests**			
Worse than expected	23 (15.3)	3 (11.5)	0.771
Better than expected	127 (84.7)	23 (88.5)	
**Self-conscious about appearance**			
Worse than expected	33 (22.1)	7 (26.9)	0.593
Better than expected	116 (77.9)	19 (73.1)	
**Sense of sexuality**			
Worse than expected	33 (24.8)	9 (36)	0.245
Better than expected	100 (75.2)	16 (64)	
**Worry/anxiety about breast cancer**			
Worse than expected	21 (14.1)	2 (7.7)	0.535
Better than expected	128 (85.9)	24 (92.3)	
Recovery from reconstruction surgery *			
Worse than expected	14 (34.1)	13 (59.1)	0.056
Better than expected	27 (65.9)	9 (40.9)	
Complications from reconstruction surgery *			
Worse than expected	11 (27.5)	12 (54.5)	0.035
Better than expected	29 (72.5)	10 (45.5)	
Filling up expanders *			
Worse than expected	11 (32.4)	11 (68.8)	0.016
Better than expected	23 (67.6)	5 (31.3)	

CPM, contralateral prophylactic mastectomy; UM, unilateral mastectomy; * only applicable to women who had reconstruction.

**Table 6:** Breast cancer knowledge, risk perception, and worry among both groups.

	UM N (%)	CPM N (%)	*p*-Value
**How much did you think that having your breast(s) removed would lower your chance of getting breast cancer in that breast or chest area in the future?**			
A lot	111 (74)	23 (88.5)	0.110
A little/Not at all	39 (26)	3 (11.5)	
**How concerned were you about being diagnosed with breast cancer in your other breast sometime in the future?**			
A lot	92 (61.3)	22 (84.6)	0.022
A little/Not at all	58 (38.7)	4 (15.4)	
**With treatment, about how many women diagnosed with early breast cancer will eventually die of breast cancer?**			
Most will die of breast cancer	1 (0.7)	0	<0.001
About half will die of breast cancer	16 (10.7)	12 (46.2)	
Most will die of something else	132 (88.6)	14 (53.8)	
**On average, which women with early breast cancer will live longer?**			
Women who have a mastectomy	17 (11.4)	0	0.029
Women who have a bilateral mastectomy	60 (40.3)	17 (65.4)	
There is no difference	72 (48.3)	9 (34.6)	
**In general, how worried are you about breast cancer now?**			
Worried	94 (62.7)	16 (61.5)	0.913
Not worried	56 (37.3)	10 (38.5)	
**Do you think the chance your cancer will come back is higher, lower or about the same as other women with early-stage breast cancer?**			
Higher	22 (14.7)	8 (30.8)	<0.001
Lower	30 (20)	12 (46.2)	
About Same	98 (65.3)	6 (23.1)	

## Data Availability

The original contributions presented in this study are included in the article/Supplementary Material. Further inquiries can be directed to the corresponding author(s).

## Supplementary Materials

The following supporting information can be downloaded at: https://www.mdpi.com/article/10.3390/medsci13040225/s1, Supplementary File S1: Decision-Making and Experiences Among Patients Who Chose Contralateral Prophylactic Mastectomy (CPM); Supplementary File S2: Decision-Making and Experiences Among Patients Who Chose Unilateral Mastectomy (UM).

## References

[1] Ferlay J., Colombet M., Soerjomataram I., Mathers C., Parkin D.M. (2019). Estimating the global cancer incidence and mortality in 2018: GLOBOCAN sources and methods. Int. J. Cancer.

[2] Ginsburg O., Yip C.H., Brooks A., Cabanes A., Caleffi M., Dunstan Yataco J.A., Gyawali B., McCormack V., McLaughlin de Anderson M., Mehrotra R. (2020). Breast cancer early detection: A phased approach to implementation. Cancer.

[3] Schnitt S.J., Moran M.S., Giuliano A.E. (2020). Lumpectomy Margins for Invasive Breast Cancer and Ductal Carcinoma in Situ: Current Guideline Recommendations, Their Implications, and Impact. J. Clin. Oncol..

[4] Fares M.Y., Salhab H.A., Khachfe H.H., Khachfe H.M. (2019). Breast Cancer Epidemiology among Lebanese Women: An 11-Year Analysis. Medicina.

[5] Gradishar W.J., Abo J., Abraham J. (2020). Breast Cancer, NCCN Evidence Blocks.

[6] Lim D.W., Metcalfe K.A., Narod S.A. (2021). Bilateral Mastectomy in Women with Unilateral Breast Cancer: A Review. JAMA Surg..

[7] Boughey J.C., Attai D.J., Chen S.L., Cody H.S., Dietz J.R., Feldman S.M., Greenberg C.C., Kass R.B., Landercasper J., Lemaine V. (2016). Contralateral Prophylactic Mastectomy (CPM) Consensus Statement from the American Society of Breast Surgeons: Data on CPM Outcomes and Risks. Ann. Surg. Oncol..

[8] Kramer I., Schaapveld M., Oldenburg H.S.A., Sonke G.S., McCool D., van Leeuwen F.E., Van de Vijver K.K., Russell N.S., Linn S.C., Siesling S. (2019). The Influence of Adjuvant Systemic Regimens on Contralateral Breast Cancer Risk and Receptor Subtype. J. Natl. Cancer Inst..

[9] Xiong Z., Yang L., Deng G., Huang X., Li X., Xie X., Wang J., Shuang Z., Wang X. (2018). Patterns of Occurrence and Outcomes of Contralateral Breast Cancer: Analysis of SEER Data. J. Clin. Med..

[10] Daly M.B., Pal T., Berry M.P., Buys S.S., Dickson P., Domchek S.M., Elkhanany A., Friedman S., Goggins M., Hutton M.L. (2021). Genetic/Familial High-Risk Assessment: Breast, Ovarian, and Pancreatic, Version 2.2021, NCCN Clinical Practice Guidelines in Oncology. J. Natl. Compr. Cancer Netw..

[11] Greener J.R., Bass S.B., Lepore S.J. (2018). Contralateral prophylactic mastectomy: A qualitative approach to exploring the decision making process. J. Psychosoc. Oncol..

[12] Hawley S.T., Jagsi R., Morrow M., Janz N.K., Hamilton A., Graff J.J., Katz S.J. (2014). Social and Clinical Determinants of Contralateral Prophylactic Mastectomy. JAMA Surg..

[13] Veronesi U., Cascinelli N., Mariani L., Greco M., Saccozzi R., Luini A., Aguilar M., Marubini E. (2002). Twenty-year follow-up of a randomized study comparing breast-conserving surgery with radical mastectomy for early breast cancer. N. Engl. J. Med..

[14] Fisher B., Anderson S., Bryant J., Margolese R.G., Deutsch M., Fisher E.R., Jeong J.H., Wolmark N. (2002). Twenty-year follow-up of a randomized trial comparing total mastectomy, lumpectomy, and lumpectomy plus irradiation for the treatment of invasive breast cancer. N. Engl. J. Med..

[15] Fayanju O.M., Stoll C.R., Fowler S., Colditz G.A., Margenthaler J.A. (2014). Contralateral prophylactic mastectomy after unilateral breast cancer: A systematic review and meta-analysis. Ann. Surg..

[16] Portschy P.R., Kuntz K.M., Tuttle T.M. (2014). Survival outcomes after contralateral prophylactic mastectomy: A decision analysis. J. Natl. Cancer Inst..

[17] Ager B., Butow P., Jansen J., Phillips K.A., Porter D. (2016). Contralateral prophylactic mastectomy (CPM): A systematic review of patient reported factors and psychological predictors influencing choice and satisfaction. Breast.

[18] Soran A., Kamali Polat A., Johnson R., McGuire K.P. (2014). Increasing trend of contralateral prophylactic mastectomy: What are the factors behind this phenomenon?. Surg. J. R. Coll. Surg. Edinb. Irel..

[19] Kurian A.W., Li Y., Hamilton A.S., Ward K.C., Hawley S.T., Morrow M., McLeod M.C., Jagsi R., Katz S.J. (2017). Gaps in Incorporating Germline Genetic Testing Into Treatment Decision-Making for Early-Stage Breast Cancer. J. Clin. Oncol. Off. J. Am. Soc. Clin. Oncol..

[20] Jagsi R., Jiang J., Momoh A.O., Alderman A., Giordano S.H., Buchholz T.A., Pierce L.J., Kronowitz S.J., Smith B.D. (2016). Complications After Mastectomy and Immediate Breast Reconstruction for Breast Cancer: A Claims-Based Analysis. Ann. Surg..

[21] Rosenberg S.M., Tracy M.S., Meyer M.E., Sepucha K., Gelber S., Hirshfield-Bartek J., Troyan S., Morrow M., Schapira L., Come S.E. (2013). Perceptions, knowledge, and satisfaction with contralateral prophylactic mastectomy among young women with breast cancer: A cross-sectional survey. Ann. Intern. Med..

[22] Jones N.B., Wilson J., Kotur L., Stephens J., Farrar W.B., Agnese D.M. (2009). Contralateral prophylactic mastectomy for unilateral breast cancer: An increasing trend at a single institution. Ann. Surg. Oncol..

[23] Stucky C.C., Gray R.J., Wasif N., Dueck A.C., Pockaj B.A. (2010). Increase in contralateral prophylactic mastectomy: Echoes of a bygone era? Surgical trends for unilateral breast cancer. Ann. Surg. Oncol..

[24] King T.A., Sakr R., Patil S., Gurevich I., Stempel M., Sampson M., Sampson M., Morrow M. (2011). Clinical management factors contribute to the decision for contralateral prophylactic mastectomy. J. Clin. Oncol. Off. J. Am. Soc. Clin. Oncol..

[25] Jeon H.J., Park H.S. (2019). Trends in contralateral prophylactic mastectomy rate according to clinicopathologic and socioeconomic status. Ann. Surg. Treat. Res..

[26] Graves K.D., Peshkin B.N., Halbert C.H., DeMarco T.A., Isaacs C., Schwartz M.D. (2007). Predictors and outcomes of contralateral prophylactic mastectomy among breast cancer survivors. Breast Cancer Res. Treat..

[27] Covelli A.M., Baxter N.N., Fitch M.I., McCready D.R., Wright F.C. (2015). ‘Taking control of cancer’: Understanding women’s choice for mastectomy. Ann. Surg. Oncol..

[28] Tollow P., Williams V.S., Harcourt D., Paraskeva N. (2019). “It felt like unfinished business, it feels like that’s finished now”: Women’s experiences of decision making around contralateral prophylactic mastectomy (CPM). Psycho-oncology.

[29] You Q., Chen K., Li Y., Lai J., Fang Y., Shen S., Liu Y., Su F., Yu F. (2018). Factors associated with the increasing trend of contralateral prophylactic mastectomy among patients with ductal carcinoma in situ: Analysis of Surveillance, Epidemiology, and End Results data. Breast.

[30] Montgomery L.L., Tran K.N., Heelan M.C., Van Zee K.J., Massie M.J., Payne D.K., Borgen P.I. (1999). Issues of regret in women with contralateral prophylactic mastectomies. Ann. Surg. Oncol..

[31] Soran A., Ibrahim A., Kanbour M., McGuire K., Balci F.L., Polat A.K., Thomas C., Bonaventura M., Ahrendt G., Johnson R. (2015). Decision making and factors influencing long-term satisfaction with prophylactic mastectomy in women with breast cancer. Am. J. Clin. Oncol..

[32] Anderson C., Islam J.Y., Elizabeth Hodgson M., Sabatino S.A., Rodriguez J.L., Lee C.N., Sandler D.P., Nichols H.B. (2017). Long-Term Satisfaction and Body Image After Contralateral Prophylactic Mastectomy. Ann. Surg. Oncol..

