# Supplementary material for: Understanding Patient Decision-Making in Breast Cancer Surgery: Risk Perception, Communication, and Psychosocial Influences
Source: Med Sci (Basel). 2025 Oct 9;13(4):225. doi: 10.3390/medsci13040225 (PMC12550996; doi:10.3390/medsci13040225)
Supplement: Supplementary file 1 [file medsci-13-00225-s001.zip › Supplementary Material 2.pdf]

Decision-Making and Experiences Among Patients Who Chose Unilateral Mastectomy (UM)

Who first brought up the idea of having your breast(s) removed?

- ☐ Myself
- ☐ Medical oncologist
- ☐ Plastic surgeon
- ☐ Cancer surgeon
- ☐ Nurse
- ☐ Genetic counselor
- ☐ Family/friend
- ☐ Another breast cancer patient
- ☐ Other (please write in): ____________________

How much did you and your doctor talk about the reasons for having your breast(s) removed?

- ☐ A lot
- ☐ A little/Not at all

How much did you and your doctor talk about the reasons not to have your breast(s) removed?

- ☐ A lot
- ☐ A little/Not at all

How much did you and your doctor talk about how you felt about the possibility that cancer might occur in the other breast?

- ☐ A lot
- ☐ A little/Not at all

Women have their breast(s) removed for many reasons. For each of the following, please indicate whether or not it was an important reason for your decision about surgery, or whether it did not apply to you.

		Yes, important	No, not at all important
a)	Desire to lower the chance of getting breast cancer in "the same breast"	☐	☐
b)	Desire to prevent breast cancer from spreading to other places in body.	☐	☐
c)	Desire to improve my survival/extend my life	☐	☐
d)	Desire for peace of mind	☐	☐
e)	Desire to follow my doctor's recommendation	☐	☐
f)	Advice from friends or family	☐	☐
g)	Have known genetic change (mutation) such as a BRCA 1 or BRCA 2 mutation	☐	☐
h)	Have a strong family history of breast cancer	☐	☐
i)	Other ____________________	☐	☐

Please review your responses to the question above. **Circle the single most important reason you had for removing your breast(s).**

How much did you think that having your breast(s) removed would lower your chance of getting breast cancer in that breast or chest area in the future?

- ☐ A lot
- ☐ A little/Not at all

How concerned were you about being diagnosed with breast cancer in your other breast sometime in the future?

- ☐ A lot
- ☐ A little/Not at all

THE NEXT FEW QUESTIONS ASK ABOUT SOME FACTS THAT DOCTORS THINK ARE IMPORTANT FOR PATIENTS TO KNOW ABOUT BREAST CANCER. THE CORRECT ANSWER TO EACH QUESTION IS BASED ON MEDICAL RESEARCH. FOR THESE QUESTIONS WE ARE INTERESTED IN WHAT YOU KNOW. PLEASE DON'T FEEL LIKE YOU HAVE TO FIND THE "RIGHT" ANSWER.

With treatment, about how many women diagnosed with early breast cancer will eventually die of breast cancer?

- ☐ Most will die of breast cancer
- ☐ About half will die of breast cancer
- ☐ Most will die of something else

On average, which women with early breast cancer will live longer?

- ☐ Women who have a mastectomy
- ☐ Women who have a bilateral mastectomy
- ☐ There is no difference

FOR THE NEXT TWO QUESTIONS, YOU MAY NOT KNOW THE EXACT NUMBER, BUT PLEASE WRITE IN YOUR BEST GUESS IN THE SPACE PROVIDED.

If 100 women with early breast cancer are treated with a single mastectomy or lumpectomy and radiation, about how many will develop breast cancer in the "other breast" in the 5 years after treatment?

___________ women out of 100

If 100 women with early breast cancer have both breasts removed, how many will have breast cancer come back in the chest wall area of the "other breast" in the five years after treatment?

____________ women out of 100

On a scale from 0 to 10, where 10 means extremely confident and 0 means not confident at all, how confident are you that the decision about removing your breast(s) was the right one for you?

- ☐ 10 Extremely confident
- ☐ 9
- ☐ 8
- ☐ 7
- ☐ 6
- ☐ 5 Somewhat confident
- ☐ 4
- ☐ 3
- ☐ 2
- ☐ 1
- ☐ 0 Not confident at all

If you could make this decision again, would you still choose to have your breast(s) removed?

- ☐ Yes, definitely
- ☐ No, probably not

For each of the following, please indicate whether you feel that your experience was better than you expected, about what you expected or worse than you expected.

	Worse than expected	Better than expected
a) Cosmetic results (the way your chest looks)	□	□
b) Pain at surgical site	□	□
c) Number of surgeries/procedures needed	□	□
d) Numbness or tingling in chest	□	□
e) Self-conscious about your appearance	□	□
f) Sense of sexuality	□	□
g) Worry or anxiety about breast cancer	□	□
h) Amount of follow up imaging or tests	□	□
If you had reconstruction, please answer the following:		
i) Recovery from reconstruction surgery	□	□
j) Complications or problems from reconstruction surgery	□	□
k) Filling up expanders	□	□

For each of the following, please mark whether or not it was true for you when you were making a choice about surgery for breast cancer.

a)	Did you feel that you knew the benefits and risks of each option?	☐ Yes	☐ No
b)	Were you clear about which benefits and risks mattered most to you?	☐ Yes	☐ No
c)	Did you have enough support and advice to make a choice?	☐ Yes	☐ No
d)	Did you feel sure about the best choice for you?	☐ Yes	☐ No

In general, how worried are you about breast cancer now?

☐ Worried
☐ Not at all worried

Do you think the chance your cancer will come back is higher, lower or about the same as other women with early-stage breast cancer?

☐ Higher
☐ Lower
☐ About same

For each of the following please mark whether or not it was an important source of information for your decision about having your breast(s) removed:

a)	Doctor(s)	☐ extremely important	☐ Very important	☐ Somewhat important	☐ Not at all important	☐ Not sure
b)	Nurse(s)	☐ extremely important	☐ Very important	☐ Somewhat important	☐ Not at all important	☐ Not sure
c)	Family and friends	☐ extremely important	☐ Very important	☐ Somewhat important	☐ Not at all important	☐ Not sure
d)	Newspapers, television, magazines	☐ extremely important	☐ Very important	☐ Somewhat important	☐ Not at all important	☐ Not sure
e)	Internet, websites	☐ extremely important	☐ Very important	☐ Somewhat important	☐ Not at all important	☐ Not sure
f)	Other (please write in): ____________________	☐ extremely important	☐ Very important	☐ Somewhat important	☐ Not at all important	☐ Not sure

THANK YOU FOR COMPLETING THIS SURVEY.
